# Human bone morphogenetic protein-2 (hBMP-2) characterization by physical–chemical, immunological and biological assays

**DOI:** 10.1186/s13568-020-0964-5

**Published:** 2020-02-17

**Authors:** Miriam Fussae Suzuki, João Ezequiel Oliveira, Renata Damiani, Eliana Rosa Lima, Kleicy Cavalcante Amaral, Anderson Maikon de Souza Santos, Geraldo Santana Magalhães, Leonardo Perez Faverani, Luis Antonio Violin Dias Pereira, Fabiana Medeiros Silva, Paolo Bartolini

**Affiliations:** 1 Biotechnology Center, Instituto de Pesquisas Energéticas e Nucleares, IPEN-CNEN/SP, Avenida Prof. Lineu Prestes 2242, Cidade Universitária, São Paulo, SP 05508-000 Brazil; 2 Biosintesis P&D, São Paulo, SP Brazil; 3 Department of Surgery and Integrated Clinic-Universidade Estadual Paulista Júlio de Mesquita Filho-UNESP, School of Dentistry, Araçatuba, SP Brazil; 4 Immunopathology Laboratory, Instituto Butantan, São Paulo, SP Brazil; 5 Department of Biochemistry and Tissue Biology, Institute of Biology, Universidade Estadual de Campinas-UNICAMP, Campinas, SP Brazil

**Keywords:** BMP-2, *Escherichia coli*-derived, CHO cell-derived, C2C12 bioassay, Efficient osteoinductor

## Abstract

Commercially available preparations of methionyl-human BMP-2 and CHO-derived hBMP-2, which belongs to the transforming growth factor β (TGF-β) superfamily, were used for a complete characterization. This protein is an extremely efficient osteoinductor that plays an important role during bone regeneration and embryonic development. Characterization was carried out via SDS-PAGE and Western blotting, followed by reversed-phase HPLC, size-exclusion HPLC and MALDI-TOF-MS. The classical in vitro bioassay, based on the induction of alkaline phosphatase activity in C2C12 cells, confirmed that hBMP-2 biological activity is mostly related to the dimeric form, being ~ 4-fold higher for the CHO-derived glycosylated form when compared with the *E. coli* counterpart. The *E. coli*-derived met-hBMP-2 has shown, by MALDI-TOF-MS, a large presence of the bioactive dimer. A more complex molecular mass (MM) distribution was found for the CHO-derived product, whose exact MM has never been reported because of its variable glycosylation. A method based on RP-HPLC was set up, allowing a quantitative and qualitative hBMP-2 determination even directly on ongoing culture media. Considering that hBMP-2 is highly unstable, presenting moreover an extremely high aggregate value, we believe that these data pave the way to a necessary characterization of this important factor when synthesized by DNA recombinant techniques in different types of hosts.

## Introduction

Human bone morphogenetic protein-2 (hBMP-2), one of the most efficient osteoinductors ever described, derives its discovery from the biological basis of bone morphogenesis, set up in the pioneering works of Urist and cols. (Quaas et al. 2018; Strates et al. 1971; Urist 1965; Urist and Strates 1971). Its final purification and characterization from demineralized bone matrix, was carried out by the Reddi laboratory (Reddi and Huggins 1972; Sampath et al. 1987; Sampath and Reddi 1981), which opened the way to its cloning and CHO-derived synthesis at the Genetics Institute of Cambridge, MA, USA (Israel et al. 1992; Wozney et al. 1988).

BMP-2 is a homodimeric cysteine-knot protein, belonging to the transforming growth factor-β (TGF-β) family, whose structure is stabilized through dimerization and by an additional intermolecular disulfide-bond (Quaas et al. 2018; Scheufler et al. 1999). Due to its unique capacity of inducing bone regeneration and ectopic bone formation in adult vertebrates, its recombinant form is a good alternative to autologous bone grafting, used in many orthopedic applications such as spinal fusions, oral surgery, bone, cartilage, tendons and ligaments repair, in general (Boden 2000; de Freitas et al. 2016; Kirker-Head 2000; Vallejo et al. 2002; Wikesjo et al. 2009).

One of the most widely used recombinant preparations of hBMP-2 is CHO-derived Infuse^®^ from Medtronic (Even et al. 2012; Ong and Bouazza-Marouf 2000), but a variety of *E. coli*-derived preparations, obtained through in vitro refolding of inclusion bodies, have also shown good biological activity (Bessho et al. 2000; Lee et al. 2011; Long et al. 2006; Quaas et al. 2018; Ruppert et al. 1996; Vallejo et al. 2002). Several in vivo and in vitro studies have also clearly demonstrated their comparable osteoinductivity and clinical efficacy (Harada et al. 2012; Jin et al. 2019; Lee et al. 2013; Yano et al. 2009).

Considering that hBMP-2 has in general an extremely high aggregate value, a careful use of its precious commercial products, also considered a type of reference preparations, is mandatory for studying its characteristics and properties and, eventually, planning alternative and more efficient synthesis processes. Our research group has therefore chosen two specific preparations: the first is an *E. coli*-derived met-hBMP-2 obtained by proprietary techniques at GenScript (Piscataway, NJ, USA), while the second is the previously mentioned CHO-derived Infuse^®^, from Medtronic (Minneapolis, MN, USA). Therefore, in the present work, the two preparations have been extensively characterized via SDS-PAGE and Western blotting, reversed-phase HPLC (RP-HPLC) and size-exclusion HPLC (HPSEC), MALDI-TOF-MS and the classical in vitro bioassay based on the induction of alkaline phosphatase activity in murine myoblastic C2C12 cells. A novel methodology based on RP-HPLC has also been validated and has shown to be able to quantitatively and qualitatively determine hBMP-2 in *E. coli* extracts even during the fermentation process.

## Materials and methods

### Commercial preparations of recombinant hBMP-2

Two commercial preparations of recombinant hBMP-2 were used: met-hBMP-2 from *E. coli* (GenScript, Piscataway, NJ, USA), a dimer of two identical proteins which was lyophilized by the manufacturer after extensive dialysis against 50 mM acetic acid and reconstituted in our laboratory in 20 mM acetic acid, and Infuse^®^, a disulfide-linked dimeric protein molecule with two major subunit species of 114 and 131 amino acids. Each subunit of the latter is glycosylated at one site with high-mannose-type glycans. Infuse^®^ is produced by Medtronic (Minneapolis, MN, USA) in a genetically engineered Chinese hamster ovary (CHO) cell line, lyophilized together with excipients by the manufacturer and reconstituted in our laboratory with sterile water, presenting then a pH of 4.5 (Medtronic Medical Information Sheet, 2015).

### SDS-PAGE and Western blotting

*E. coli*-derived met-hBMP-2 (GenScript) and CHO-derived hBMP-2 (Infuse^®^) were analyzed under reducing and non-reducing conditions (Soares et al. 2000). Coomassie Brilliant Blue G-250 was used for the staining. For Western blotting, the semi-dry transfer technique was utilized on a nitrocellulose membrane, with anti-hBMP-2 affinity-purified rabbit IgG (1:2000) (Biovision, Milpitas, CA, USA) and goat anti-rabbit IgG conjugated to horseradish peroxidase (1:5000). Protein visualization was performed with Luminata Forte (Merck, Burlington, MA, USA) on X-ray film (CL-Xposure™ Film, Thermo Scientific, Rockford, IL, USA).

### High-performance size-exclusion chromatography (HPSEC)

HPSEC for analytical and preparative purposes was carried out on a G2000 SW column (60 cm × 7.5 mm I.D., particle size of 10 μm and pore size of 125 Å) from Tosoh Bioscience (Montgomeryville, PA, USA), connected to a Shimadzu Model SCL-10 A HPLC apparatus. Detection was by UV absorbance at 220 nm with a flow rate of 1.0 mL/min, employing 0.15 M NaCl in 0.02 M sodium phosphate buffer, pH 7.0, as the mobile phase.

### Analytical reversed-phase high-performance liquid chromatography (RP-HPLC)

RP-HPLC was carried out with a Jupiter C4 column (25 cm × 4.6 mm I.D., 5 μm particle size and 300 Å pore size), connected to a 4 × 3 mm guard column cartridge (Phenomenex, Torrance, CA, USA), inserted into a Shimadzu HPLC apparatus. Chromatography was carried out at 30 °C with UV absorbance detection at a wavelength of 220 nm. Two solutions were utilized: solution A being TFA 1:1000 in H_2_O and solution B, 10% A in acetonitrile. For hBMP-2 elution a linear gradient from 30% B (v/v) to 60% B (v/v) over 30 min was used, followed by an isocratic elution step with 60% B for 5 min.

### Mass spectrometry for molecular mass determination

The exact molecular mass determination of rec-hBMP-2, either of *E. coli* or of CHO origin, and of its different components, was performed via MALDI-TOF-MS at Asparia Glycomics SL (Donostia, San Sebastián, Spain). A diluted protein or glycoprotein solution (1:5, 1:10 and 1:20 from a 1 mg/mL solution) was mixed 1:1 with MALDI matrix solution (sinapinic acid 7 mg/mL in 0.1% TFA and 50% acetonitrile) and spotted directly to the MALDI plate (1 μL). The analysis was carried out in linear positive mode, in the range of 5000–40,000 Da in UltrafleXtreme MALDI-TOF-MS equipment (Bruker Daltonics, Bremen, Germany). The Open Source Mass Spectrometry tool data processing software was used for increasing resolution analysis.

### In vitro hBMP-2 bioassay in C2C12 cells

The biological activity of hBMP-2 was determined via induction of alkaline phosphatase activity in murine myoblastic C2C12 cells (Kirsch et al. 2000). Briefly, C2C12 cells (ATCC^®^—CRL-1772) were grown in DMEM with 2 mM l-glutamine, 0.1 mM non-essential amino acids, 1 mM sodium pyruvate and 10% fetal bovine serum at 37 °C and 5% CO_2_. One hundred microliters of C2C12 cells (3 × 10^5^ cells/mL), were added to a 96 well plate, the medium being replaced after 24 h with fresh medium, 2% calf serum, and with different concentration of hBMP-2, each point being determined by intra-assay duplicate. After 72 h cells were lysed in 0.2 mL buffer A (0.1 M glycerol, pH 9.6, 1% NP-40, 1 mM MgCl_2_ and 1 mM ZnCl_2_). Then, 50 μL of cell lysates were mixed with 150 μL of 0.3 mM p-nitrophenyl-phosphate (Sigma) in buffer A and incubated at 37 °C for 30 min. The alkaline phosphatase activity was determined using a Multiskan EX Microplate Reader (Thermo Electron Corporation, Beverly, MA, USA), reading the absorbance at 405 nm.

## Results

**Fig. 1:**
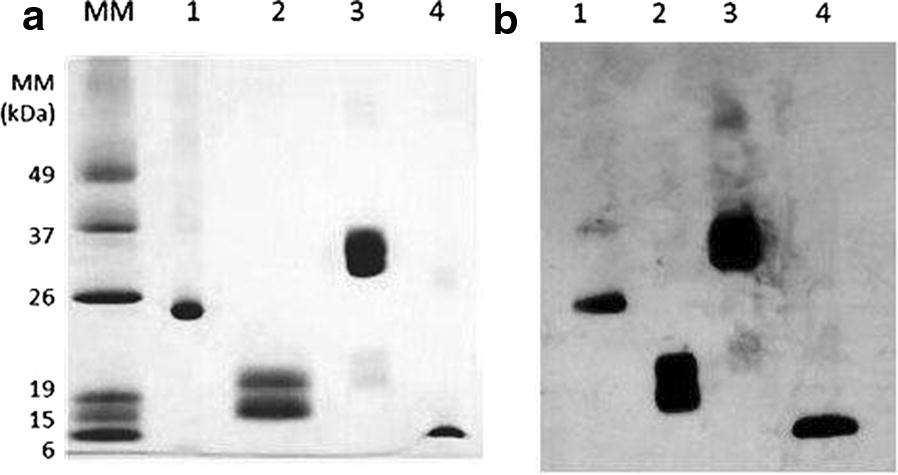
**a** SDS-PAGE analysis run in 15% acrylamide gel, stained with Coomassie Blue G, of: (MM) molecular mass markers; (1) *E. coli*-derived met-hBMP-2 (GenScript) 1 µg, run under non-reducing conditions; (2) CHO-derived hBMP-2 (Infuse^®^ from Medtronic) 1 µg, run under reducing conditions; (3) same sample as in 2, run under non-reducing conditions; (4) same sample as in 1, run under reducing conditions. **b** Western blot analysis of: (1) *E. coli*-derived met-hBMP-2, 0.1 µg, run under non-reducing conditions; (2) CHO-derived hBMP-2 (Infuse^®^), 0.25 µg, run under reducing conditions; (3) same sample as in 2, run under non-reducing conditions; (4) same sample as in 1, run under reducing conditions

A SDS-PAGE analysis of *E. coli*-derived met-hBMP-2 showed a unique dimeric electrophoretic band (~ 24,000 Da) which is then totally converted into monomer under reducing conditions (Fig. 1). It is interesting to observe the absolute absence of monomeric form in this GenScript product.

The *E. coli*-derived preparation was compared, via SDS-PAGE and Western blotting, to CHO-derived hBMP-2 (Infuse^®^). One can observe here that Infuse^®^ has a wider and higher MM band under non-reducing conditions, probably due to the presence of differently glycosylated dimeric components that are then reduced to monomeric, glycosylated forms. In the same figure, we can also see the Western blotting of the same preparation with a small presence of highly polymeric forms that were not detected by regular SDS-PAGE.

**Fig. 2:**
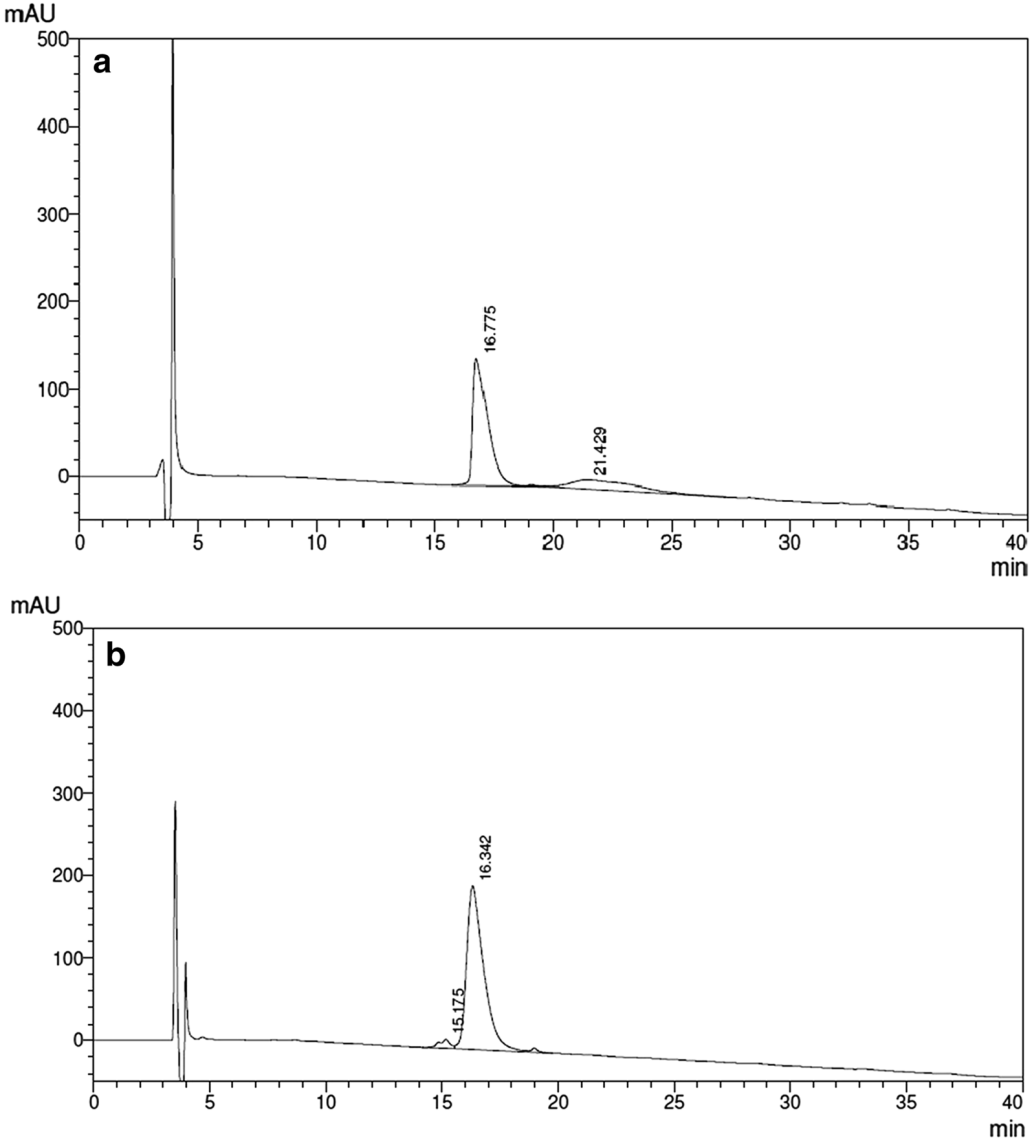
Reversed-phase high-performance liquid chromatography (RP-HPLC) of: **a***E. coli*-derived met-hBMP-2 (GenScript), 10 µg; **b** CHO-derived hBMP-2 (Infuse^®^), 10 µg

RP-HPLC analysis detected a type of protein alteration due to a peak with retention time (t_R_) = 21.42 min occurring in met-hBMP-2 and that was found to increase during storage at − 20 °C. Major peaks, with similar retention times (16.77 and 16.34 min) and, consequently, with similar hydrophobicity, are observed in both preparations (Fig. 2).

**Fig. 3:**
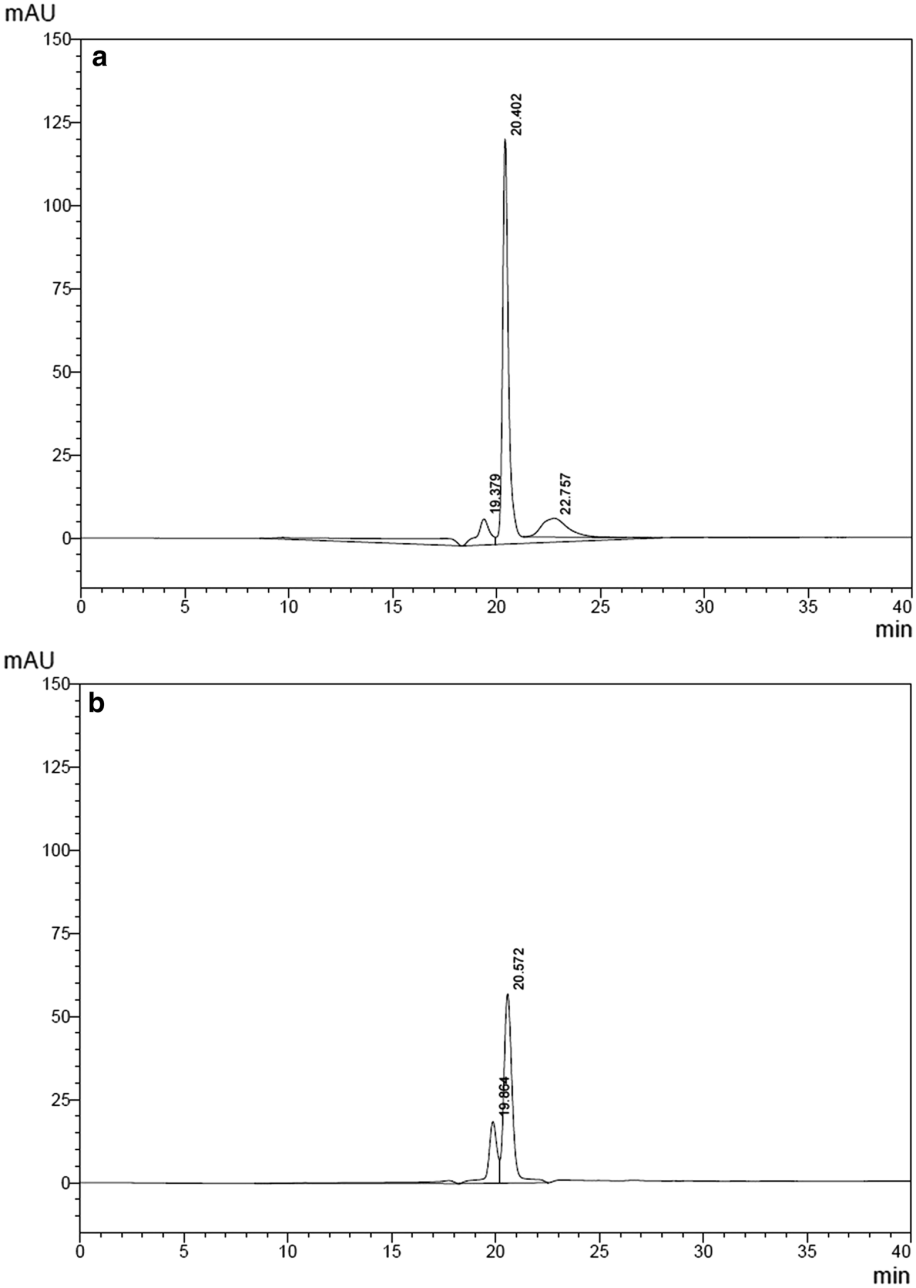
High-performance size-exclusion chromatography (HPSEC) of: **a***E. coli*-derived hBMP-2 (GenScript), 10 µg; **b** CHO-derived hBMP-2 (Infuse^®^), 10 µg

These data are confirmed by HPSEC analysis (Fig. 3), in which Infuse^®^, besides the main peak (t_R_ = 20.57 min), presented a small fraction of approximately 10% (t_R_ = 19.66 min), with a higher MM, probably due to a glycosylated form, as will be shown below by MALDI-TOF-MS analysis.

**Fig. 4:**
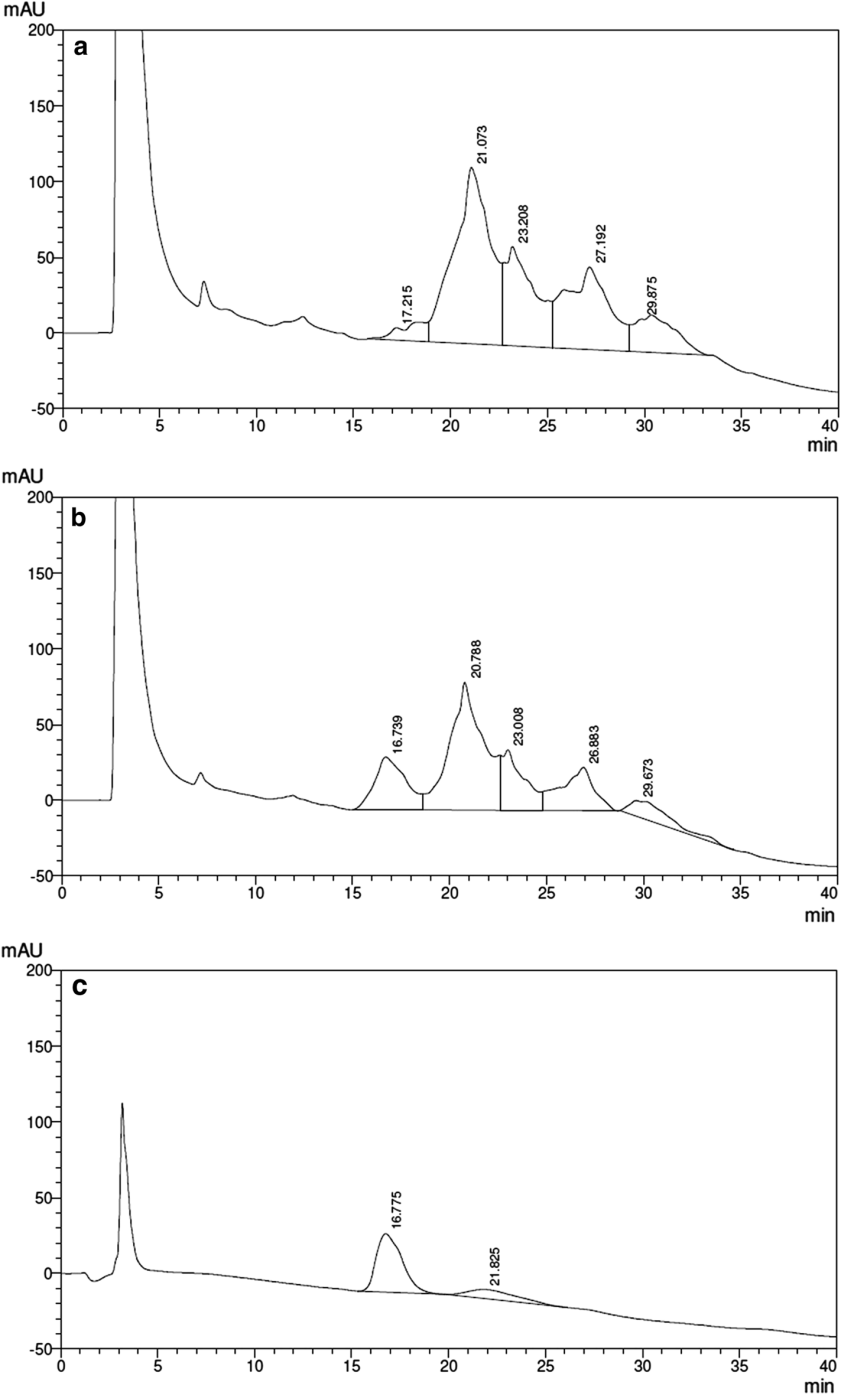
RP-HPLC quantitative and qualitative analysis of *E. coli*-derived hBMP-2: **a** hBMP-2-containing conditioned medium, 100 μL; **b** same conditioned medium to which a known amount (10 μg) of the GenScript reference preparation was added; **c** GenScript reference preparation (10 µg), used for quantitative determination

The set up method for qualitative and quantitative RP-HPLC analysis of hBMP-2, even during *E. coli* fermentation, is presented in Fig. 4, where a conditioned *E. coli* medium (Fig. 4a) is compared to the same medium to which a known amount of the GenScript reference is added (Fig. 4b) and to the pure GenScript preparation (Fig. 4c). One can observe that the added GenScript preparation appears in the same position of previously synthesized hBMP-2. According to this quantitative analysis, the medium shown in Fig. 4a contains ~ 18 μg/mL of hBMP-2.

**Fig. 5:**
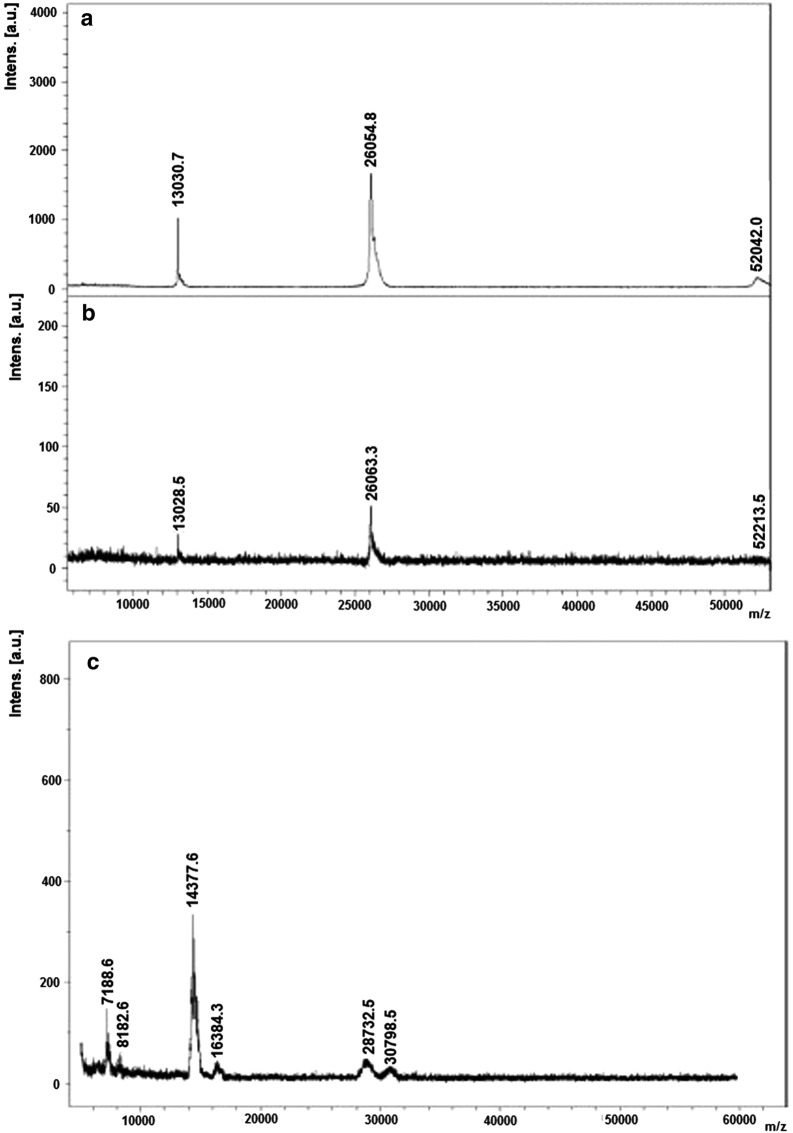
MALDI-TOF-MS for exact molecular mass (MM) determination of: **a***E. coli*-derived met-hBMP-2 from GenScript (theoretical MM = 13,036 Da), lyophilized after extensive dialysis against 50 mM acetic acid; **b** same as in “**a**”, reconstituted with 20 mM acetic acid as recommended; **c** CHO-derived hBMP-2 (Infuse^®^)

MALDI-TOF-MS for exact MM determination of both reference preparations is presented in Fig. 5. We can observe the very high accuracy of the method, considering that the theoretical MM of dimeric met-hBMP-2 is 26,072 Da, while the determined mass is 26,054 Da, showing a difference of 0.07% only, either without or with acetic acid addition in its reconstitution, as recommended by the manufacturer. The presence of a small amount of monomeric form can also be observed. In the case of Infuse^®^, not knowing the exact theoretical MM because of possible variable glycosylation, we can see the presence of four major forms, with 14,377 Da; 16,384 Da; 28,732 Da and 30,798 Da. Knowing that the theoretical protein backbone of authentic monomeric hBMP-2 (i.e. without initial Met and with a total of 114 AA) has a MM of 12,905 Da, we can deduce that the 14,377 Da form (that we will call A) can be the 114 AA monomer with 10.2% carbohydrate moiety, while the 28,732 Da form corresponds to the glycosylated dimer. Following a similar scheme and knowing from manufacturer’s data (Medical Information Sheet, Medtronic, Memphis, TN, USA) that Infuse^®^, besides the 114 AA, also contains a 131 AA form, we can calculate that this second monomeric peak of 131 AA and 16,384 Da (called B and having a protein backbone of ~ 14,800 Da) would correspond to a similarly glycosylated protein with ~ 9.6% carbohydrate. With basis on these assumptions, the 28,732 Da peak would correspond to a dimer of A, while the 30,798 Da peak, almost exactly to A + B, showing only a 0.12% difference between calculated and observed mass. Considering what suggested by Israel et al. (Israel et al. 1992), who also confirmed the presence, in mature CHO-derived hBMP-2, of a ~ 30 kDa homodimer, the monomeric forms of 14.4 kDa and 16.4 kDa are probably variants which only differ by proteolytic processing at their amino termini.

**Fig. 6:**
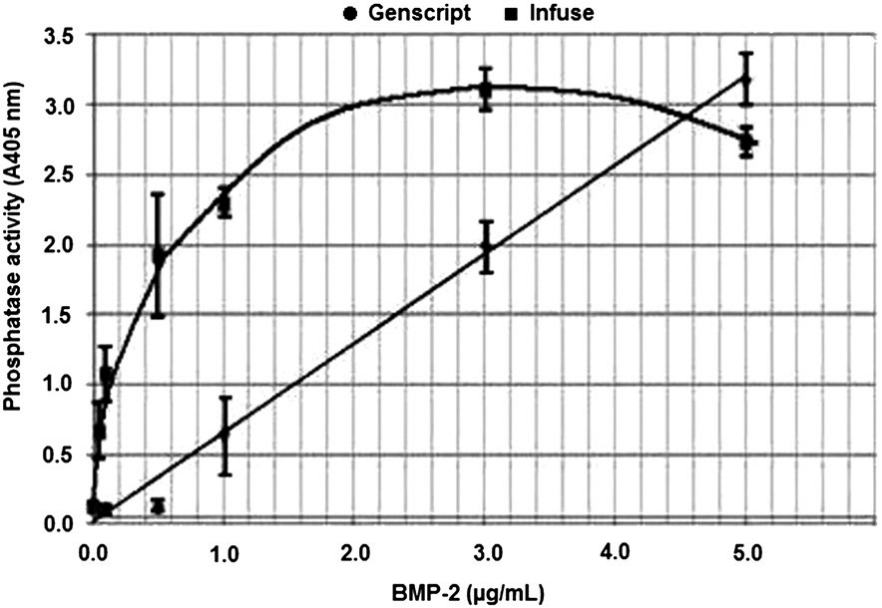
Biological activity determination of hBMP-2 via induction of alkaline phosphatase activity in murine myoblastic C2C12 cells, reading at 405 nm after 72 h culture. Equations of the assay curves: for *E. coli*-derived hBMP-2 (GenScript): ${\text{Y}}_{{{\text{A4}}0 5}} = 0. 6 3 7 {\text{X}}_{{\upmu{\text{g}}/{\text{mL}}}} {-}0.0 1 1\left( {{\text{r}} = 0. 9 9 7;{\text{n}} = 5;{\text{P}} < 0.00 1} \right);{\text{ED}}_{ 50} = 2. 5 { }\upmu{\text{g}}$; for CHO-derived hBMP-2 (Infuse^®^): ${\text{Y}}_{{{\text{A4}}0 5}} = 0. 6 8 2+ 1. 6 9 5 {\text{X}}_{{\upmu{\text{g}}/{\text{mL}}}} + 0. 2 60{\text{X}}^{ 2}_{{\upmu{\text{g}}/{\text{mL}}}} \left( {{\text{r}} = 0. 9 5 1;{\text{n}} = 7;{\text{P}} < 0.00 1} \right);{\text{ED}}_{ 50} = 0. 4 { }\upmu{\text{g}}$

We consider the in vitro bioassay based on the induction of alkaline phosphatase activity in murine myoblastic C2C12 cells as the “almost final” biological response for hBMP-2 activity. We call it “almost final” because we particularly praise, in this respect, a type of in vivo bioassay carried out in rat calvarial critical-size defects as the one described by Nakamura et al. (Nakamura et al. 2017). The C2C12 in vitro assay, however, is almost final because it is hard to speculate that a good activity in this assay will not provide activity in the in vivo test. On the other hand, the mentioned in vitro assay has been described and utilized by several authors (Kirsch et al. 2000; Long et al. 2006; Quaas et al. 2018; Vallejo et al. 2002). In Fig. 6, in fact, we are showing the curve carried out in our laboratory with the use of the GenScript preparation. The curve inclination, which is directly related to the potency of the product, was 0.637 μg/mL/A_405_ in our hands and 0.533 µg/mL/A_405_ (whole equation: Y_A405_ = 0.533X_µg/mL_ +0.024; r = 0.991; n = 4; P < 0.01) in the case of Vallejo et al. analogous curve (Vallejo et al. 2002), providing a similar potency and, in both cases, highly significant statistical parameters. Surprisingly, in our hands, the Infuse^®^ curve did not show a linear correlation but a clear parabolic function, whose equation and ED_50_ ratio indicate an activity that is ~ 4-fold higher than that shown by the GenScript preparation.

In Table 1, the in vitro bioassays carried out on CHO-derived and on *E. coli*-derived hBMP-2 all along the present study, have been compared with basis on two widely used parameters for relative potency determination: either the slope of the dose–response curve or the effective dose fifty (ED_50_) ratios. The two methods indicated, with an acceptable precision, that the CHO-derived product has indeed a potency 3.4- to 4.6-fold higher than the *E. coli*-derived preparation, in this in vitro bioassay. Unfortunately, a higher inter-assay precision was not easy to obtain, because of the high instability and extremely high cost of these preparations, which does not facilitate the repeated use of recently dissolved products.

**Table 1:** Comparison between the “in vitro” biological activity of CHO-derived (Infuse) and of *E. coli*-derived (GenScript) hBMP-2 preparations

Assay #	*E. coli*-derived hBMP-2		CHO-derived hBMP-2		Relative potency of CHO-derived hBMP-2 with basis on:	
	Slope^a^ (A_405_/µg/mL)	ED_50_ (µg/mL)	Slope^a^ (A_405_/µg/mL)	ED_50_ (µg/mL)	Slope (ratio)	ED_50_ (ratio)
1	0.637	2.50	3.081	0.40	4.8	6.2
2	0.495	1.15	3.147	0.48	6.3	2.4
3	0.543	1.18	2.056	0.40	3.8	3.0
4	0.565	1.47	2.403	0.50	4.2	2.9
5	0.704	1.23	2.746	0.47	3.9	2.6
Mean ± SD	0.589 ± 0.082	1.51 ± 0.57	2.687 ± 0.46	0.45 ± 0.047	4.60 ± 1.03	3.42 ± 1.57
CV (%)	14	38	17	10	22	46

^a^Slope of the dose–response curve: Y_A405_ = a X_µg/mL_ + b. In the case of CHO-derived hBMP-2 the equations were calculated with basis on the initial linear region of the dose–response curve

## Discussion

Two of the most widely used preparations of hBMP-2, one *E. coli*-derived and the other CHO-derived, have been compared via physical–chemical, immunological and in vitro biological assays. As already mentioned, these are considered reference preparations and there are some difficulties associated with their use, especially due to their instability.

The *E. coli*-derived preparation (met-hBMP-2 from GenScript) is declared stable up to 6 months at − 80 °C in lyophilized form from date of receipt and, upon reconstitution, only up to 2 weeks at 4 °C, or up to 3 months at − 20 °C. The CHO-derived preparation (Infuse^®^ from Medtronic) seems somehow more stable. Its validity, in lyophilized form is declared for approximately 1 year while, upon reconstitution, its immediate use together with the provided collagen sponge is recommended. The constant and repeated use of these working reference preparations is, therefore, quite unpractical. We believe, nonetheless, that our study was quite useful. SDS-PAGE and Western blotting analysis confirmed the prevalent presence of dimeric forms in both preparations. As expected, immunological activity was also present in the monomeric forms and in minor amounts of polymeric forms.

RP-HPLC and HPSEC revealed some alterations in both preparations due to storage, while the latter methodology was not so efficient, at least in our hands, to detect an increased molecular mass of approximately 10%, in the case of CHO-derived glycosylated preparations. RP-HPLC, moreover, was tested and validated as a quite useful approach for quantitative and qualitative analysis of hBMP-2 in its pure form and even directly in culture, confirming the experience of our research group in setting up analogous methodologies (Dalmora et al. 1997; Dias et al. 2018; Oliveira et al. 2003; Soares et al. 2002).

MALDI-TOF-MS molecular mass determination was of great help, providing an accurate method for checking the molecular distribution of the two preparations. Met-hBMP-2 confirmed the accuracy of this methodology while the analysis of Infuse^®^ revealed what molecular forms we are dealing with. The definition, reported in the “Medtronic Medical Information Sheet”, that Infuse^®^ is a disulfide-linked dimeric protein molecule with two major subunit species of 114 and 131 amino acids and a single glycosylation site, has been confirmed with more details in Fig. 5c. This allowed also a calculation that ~ 10% of carbohydrate moiety is due to the mentioned single glycosylation site, with probably the presence of complex and high-mannose type *N*-glycan (Israel et al. 1992). It is known that MALDI-TOF-MS analysis is not strictly quantitative and therefore one can only observe that the Infuse^®^ preparation seems rich in monomeric forms, but this could also be due to artefacts related with the specific analytical technique.

The C2C12 in vitro bioassay has demonstrated a good accuracy and inter-laboratory reproducibility, through the comparison of two curves obtained with similar *E. coli*-derived hBMP-2 preparation: a published curve (Vallejo et al. 2002) and the present, carried out in our laboratory with the use of the GenScript preparation. A sound comparison, carried out with basis on five different experiments, shown in Table 1, has proved that Infuse^®^ has a much higher in vitro bioactivity than the GenScript preparation, at least at lower doses, while at higher doses (~ 5 μg/mL) a similar potency is attained. We believe that the Nakamura et al. (Nakamura et al. 2017) approach, used to compare in vivo the osteoinductive potential of two hBMPs (hBMP-2 versus hBMP-9) by treating calvarial critical-size defects in rats, will be very useful for the purpose of studying and defining the in vivo potency of recombinant hBMP-2 preparations of different origins. It will be possible, moreover, to characterize new preparations at the molecular level, with basis on the present data and methodologies.

## Data Availability

Not applicable.
